# Empirical Research on Decoupling Relationship between Energy-Related Carbon Emission and Economic Growth in Guangdong Province Based on Extended Kaya Identity

**DOI:** 10.1155/2014/782750

**Published:** 2014-03-23

**Authors:** Wenxiu Wang, Yaoqiu Kuang, Ningsheng Huang, Daiqing Zhao

**Affiliations:** ^1^Guangzhou Institute of Energy Conversion, Chinese Academy of Sciences, Guangzhou, Guangdong 510640, China; ^2^Sustainable Development Research Center, Guangzhou Institute of Geochemistry, Chinese Academy of Sciences, Guangzhou, Guangdong 510640, China; ^3^University of Chinese Academy of Sciences, Beijing 100049, China

## Abstract

The decoupling elasticity decomposition quantitative model of energy-related carbon emission in Guangdong is established based on the extended Kaya identity and Tapio decoupling model for the first time, to explore the decoupling relationship and its internal mechanism between energy-related carbon emission and economic growth in Guangdong. Main results are as follows. (1) Total production energy-related carbon emissions in Guangdong increase from 4128 × 10^4^ tC in 1995 to 14396 × 10^4^ tC in 2011. Decoupling elasticity values of energy-related carbon emission and economic growth increase from 0.53 in 1996 to 0.85 in 2011, and its decoupling state turns from weak decoupling in 1996–2004 to expansive coupling in 2005–2011. (2) Land economic output and energy intensity are the first inhibiting factor and the first promoting factor to energy-related carbon emission decoupling from economic growth, respectively. The development speeds of land urbanization and population urbanization, especially land urbanization, play decisive roles in the change of total decoupling elasticity values. (3) Guangdong can realize decoupling of energy-related carbon emission from economic growth effectively by adjusting the energy mix and industrial structure, coordinating the development speed of land urbanization and population urbanization effectively, and strengthening the construction of carbon sink.

## 1. Introduction

IPCC Fourth Assessment Report (AR4, 2007) indicates that the increase of global greenhouse gas mainly results from the burning of fossil energy. The statistical data show that the greenhouse gas emission resulting from human production and daily activities accounts for more than 90% of the total amount of global greenhouse gas emission due to large amounts of fossil fuels since the industrial revolution [1]. The international community has reached the agreement that the continuous carbon emission reduction is an important measure to tackle the climate change positively [2]. The energy-related carbon emission in China has accounted for a large part of the world total amounts. The statistical data from IEA [3] list the energy-related carbon emission data from 1971–2010 around the world. The data indicate that the CO_2_ emission in USA was top one in the world from 1971 to 2006. CO_2_ emission amount is 6.037 billion ton in China and 5.852 billion ton in the USA in 2007 when China surpassed the United States for the first time and became the world number 1 energy-related carbon emission country. Since then, energy-related carbon emissions of China show strong growth momentum, and the CO_2_ emission amount keeps number 1 in the world. Till 2010, China CO_2_ emission amount was 7.669 billion ton, accounting for 24.66% of the world energy CO_2_ emission. China has played an important role in the issue of world carbon emission and been undertaking the increasing emission reduction stresses in international community. Our government promised to reduce  CO_2_ emission per unit GDP by 40%–45% in 2020 compared to that in 2005 in Copenhagen Climate Change Conference in late 2009, which had been already incorporated into the medium- and long-term planning program of national economic and social development as a binding indicator. Therefore, exploring the high-efficient carbon reduction measures and approaches, fulfilling the carbon reduction task effectively and realizing the low carbon economy are our main challenges.

**Figure 1:**
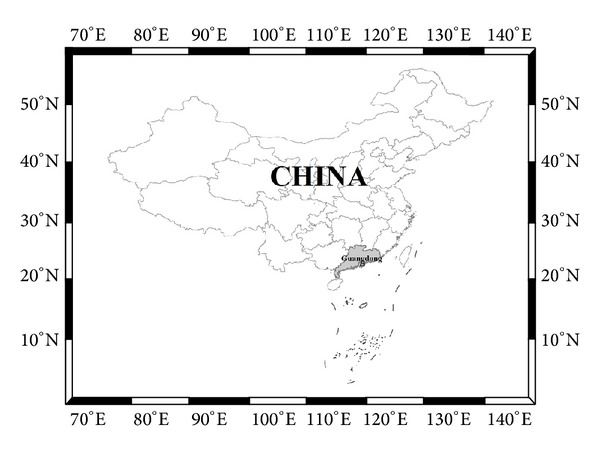
Geographic location of Guangdong in China.

Guangdong, located in the subtropical part of southern China mainland (Figure 1), between latitude 20°13′–25°31′N and longitude 109°39′–117°19′E, is one of the regions which have the most abundant light, heat, and water resources in China. It is the largest province in economy and population and urbanization in China, its gross domestic product (GDP), and permanent population and urbanization rate reached 5321 billion yuans and 105.05 million persons and 66.5% in 2011, respectively. It is also one of the largest provinces in energy consumption and its total energy consumption reached 284.8 million ton standard coal equivalent (tsce) in 2011, only behind Shandong and Hebei. The National Development and Reform Commission listed Guangdong as the national low-carbon pilot regions in 2010. The national “12th five-year plan” requires the energy consumption per unit GDP of Guangdong cut down by 18% in 2015 compared to that in 2010 while CO_2_ emission per unit GDP cut down by 19.5%. Guangdong faces huge stresses in emission reduction and needs practical and effective carbon emission reduction strategies to promote low-carbon province construction.

Many researches show that the carbon emission has close relationship with the economic growth [4–12]. In the long run, the course of moving towards low carbon economy for a country is to realize decoupling of the carbon emission from economic growth gradually. Therefore, the final method to realize the high-efficient carbon emission reduction and low carbon economy is to weaken or break the link between carbon emission and economic growth.

The word “decoupling” is firstly used in physics used to eliminate the interrelation between two or more physical quantities and response relationship [13]. Organization for Economic Cooperation and Development (OECD) first proposes the concept of decoupling and divides it into absolute decoupling and relative decoupling, which contributes to the theory research into decoupling [14]. Tapio introduces the elasticity method to decoupling research, which further develops and improves the decoupling theory [15, 16]. With the increasing aggravation of resource shortage, environmental pollution and ecological damage resulting from economic growth in 20th century, the “decoupling” idea is introduced to research into economic growth and resource shortage and greenhouse gas emission by some scholars to realize the breakdown of coupling relationship between expected variables (e.g., economic growth) and nonexpected variables (e.g., resource investment or greenhouse gas emission).

In the field of carbon emission reduction, Tapio [15] first uses the decoupling elasticity method to research into the decoupling situations between traffic volumes and greenhouse gas emission and economic growth of European transportation. Zhuang [17] researched into decoupling index of CO_2_ emission and economic growth in Taiwan. Zhuang [18] applies Tapio decoupling index to analyze the decoupling situations in different periods in global 20 greenhouse gas emission countries including China. Gray et al. [19] researched into the decoupling situations between traffic volumes and CO_2_ emission and economic growth in Scotland. Li and Qing [20] apply OECD decoupling index and Tapio decoupling index to analyze the relationship between industrial added value and energy consumption investment and CO_2_ emission in Shanxi Industrial Department.

Overall, most of foreign and domestic scholars mainly research into measuring decupling index of carbon emission and economic growth, while few of them research into the mechanism of changes of decoupling index and its decoupling state. There are only Zhao and Li [16], Wang et al. [21], and Wang et al. [22] who have made such relative research in our county. There is no research on the decoupling relationship between energy-related carbon emission and economic growth in Guangdong, and no research takes urbanization into account. In view of this, decoupling elasticity decomposition quantitative model of energy-related carbon emission is established by combing influence factors decomposition model of energy-related carbon emission, which is based on basic principle of Kaya model, with Tapio decoupling model in this paper. The main purpose is to explore the decoupling relationship and internal mechanism between energy-related carbon emission and economic growth,to seek the key influence factors of decoupling and put forward to targeted policy recommendations to realize decoupling of carbon emissions form economic growth. Provide information support and decision basis to promoting low carbon work for government of Guangdong province and provide empirical bases for national low carbon economy development.

Production sector is the main source of energy-related carbon emission and it participates in the creation of the GDP and household is not involved in the creation of the GDP, so the energy-related carbon emission in this paper only refers to the production energy-related carbon emission, not including household energy-related carbon emission.

## 2. Methods and Data Sources

### 2.1. Calculation of Energy-Related Carbon Emission

Production energy consumption refers to energy consumption by the three strata of industry.

Farming, forestry, animal husbandry, fishery, and water conservancy belong to the primary industry. Industry and construction belong to the secondary industry. Transport, storage, postal and telecommunication services, wholesale and retail trade and catering services, and others belong to the tertiary industry. Among them, energy consumption by the industry sector includes the end-use energy consumption by industry sector and energy consumption by production of thermal power and heat power. There are 17 types of energy, including coal, crude oil, natural gas, and other fossil fuels and their products, according to* Energy Balance Sheet of Guangdong Province *in* China Energy Statistical Yearbook*. Energy-related carbon emissions are calculated as follows:

(1) $$\begin{array}{c} C=\underset{i}{\sum }\underset{j}{\sum }C_{ij}=\underset{i}{\sum }\underset{j}{\sum }E_{ij}\times f_{j}, \end{array}$$

where *C* is carbon emissions from energy consumption, *i* is the type of industry, *j* is the type of energy, *C*_*ij*_ represent carbon emissions of energy *j* in industry *i*, *E*_*ij*_ represent consumption of energy *j* in industry *i*, and *f*_*j*_ is carbon emission coefficient of energy *j*. Carbon emission coefficients of different kinds of energy can be seen in Table 1.

### 2.2. Establishment of Decoupling Elasticity Decomposition Quantitative Model of Energy-Related Carbon Emission 

#### 2.2.1. Decoupling Model of Carbon Emission

There are two kinds of carbon emission decoupling models, that is, OECD decoupling model and Tapio decoupling model. The formula of OECD decoupling model is as follows:

(2) $$\begin{array}{c} D_{\text{oecd}}=1-\frac{{\left(C/\text{GDP}\right)}_{T}}{{\left(C/\text{GDP}\right)}_{0}}, \end{array}$$

where *C* is carbon emission, GDP is gross domestic product. The subscript 0 is base time and *T* is final time.

The formula of Tapio decoupling model is as follows:

(3) $$\begin{array}{c} D_{t}=\frac{\Delta C/C}{\Delta \text{GDP}/\text{GDP}}, \end{array}$$

where *C* is the carbon emission of current year, Δ*C* is the change amount of carbon emission on current time compared with the base time, GDP is the gross domestic product in current year, and ΔGDP is the change amount of GDP on current time compared with the base time. Tapio [15] defines 8 decoupling states according to the decoupling elasticity value; see Table 2.

Many scholars find the advantages of Tapio decoupling model which cannot be surpassed by OECD decoupling model through empirical verification and comparison [16]. So this paper applies Tapio decoupling model when establishing decoupling elasticity decomposition quantitative model of energy-related carbon emission.

#### 2.2.2. Decomposition Model of Energy-Related Carbon Emission Based on Kaya Identity

Kaya identity is first put forward by Japanese scholars Kaya [23, 24]. Kaya identity established the relationship between economy, policy, population, and CO_2_ emission caused by the human activity. It is widely used in the field of energy economy and environmental economy for its simple structure and easy to be operated, although its policy implication has some limitations [25]. In this paper, we establish decomposition model of energy-related carbon emission only to get change amount of carbon emission of each decomposition factor and use them in Tapio decoupling model, so limitations of Kaya identity are eliminated.

In recent years, with the acceleration of urbanization progress, the relation between urbanization and carbon emission has generally been the hot topic for scholars [26–28]. There are no researches incorporating the urbanization index into extended Kaya model in those references [29–33]. As the leading province for the reform and opening up, development model and features and speed of urbanization of Guangdong are typical. Therefore, the urbanization indexes are incorporated into decomposition model of energy-related carbon emission to explore the role of urbanization in the process of decoupling of carbon emission from economic growth. The results will provide important significance for low carbon urbanization development of our country.

The decomposition model of energy-related carbon emission is established as follows according to the basic principle of Kaya model:

(4) C=∑i∑j(CijPEij·PEijPEi·PEiGDPi·GDPiGDP   ·GDPS·SSu·SuPu·PuP·P)=∑i∑j(fij·mij·di·si·g·l·r·h·p),

where GDP is the gross domestic product. *i* is types of industrial, *j* is type of energy, *C*_*ij*_ is the carbon emission generated by *j* energy in *i* industry, *PE*_*ij*_ is the consumption of *j* energy in the *i* industry, *PE*_*i*_ is the energy consumption of *i* industry, GDP_*i*_ is the added value of *i* industry, *S* is land area, *S*_*u*_ is urban construction land area, *P*_*u*_ is nonagricultural population, and *P* is total population with residence registration. *f*_*ij*_ is coefficient of energy-related carbon emission; *m*_*ij*_ is the proportion of *j* energy in the energy consumption of *i* industry, that is, energy mix; *d*_*i*_ is the energy consumption of per unit GDP of the *i* industry, that is, energy intensity; *s*_*i*_ is the proportion of GDP of *i* industry in total GDP, that is, industrial structure; *g* is the GDP of per unit land area, that is, land economic output; *l* is the reciprocal of land urbanization rate, so its change can express the change of land urbanization indirectly; *h* is the nonagricultural rate of population and it is an important index to measure the urbanization level and we take it as population urbanization; *r* is the urban area of per capita and is the result of combined action of land urbanization and population urbanization; *p* has the same mean with *P*.

The Logarithmic Mean Divisia Index (LMDI) method is widely used in decomposition of factors affecting energy-related carbon emission for that it can satisfy the requirement of factor reversible and the residual item eliminated, which makes the model more convincing [34]. So LMDI method is also used in this paper.

Take 1995 as the base time, set the increment of carbon emission as *C*_0_ in 1995 and *C*_*T*_ in *T* year; there is

(5) $$\begin{array}{c} \Delta C=C_{T}-C_{0}. \end{array}$$

The expressions for the contribution values of the decomposed factors of the energy-related carbon emissions from the productive sector are as follows:

(6) $$\begin{array}{c} \Delta C_{f}=\underset{i}{\sum }\underset{j}{\sum }\alpha \ln\frac{{F^{T}}_{ij}}{F_{ij}^{0}},\\ \Delta C_{m}=\underset{i}{\sum }\underset{j}{\sum }\alpha \ln\frac{{M^{T}}_{ij}}{M_{ij}^{0}},\\ \Delta C_{d}=\underset{i}{\sum }\underset{j}{\sum }\alpha \ln\frac{D_{i}^{T}}{D_{i}^{0}},\\ \Delta C_{s}=\underset{i}{\sum }\underset{j}{\sum }\alpha \ln\frac{S_{i}^{T}}{S_{i}^{0}},\\ \Delta C_{g}=\underset{i}{\sum }\underset{j}{\sum }\alpha \ln\frac{G^{T}}{G^{0}},\\ \Delta C_{l}=\underset{i}{\sum }\underset{j}{\sum }\alpha \ln\frac{L^{T}}{L^{0}},\\ \Delta C_{r}=\underset{i}{\sum }\underset{j}{\sum }\alpha \ln\frac{R^{T}}{R^{0}},\\ \Delta C_{h}=\underset{i}{\sum }\underset{j}{\sum }\alpha \ln\frac{H^{T}}{H^{0}},\\ \Delta C_{p}=\underset{i}{\sum }\underset{j}{\sum }\alpha \ln\frac{P^{T}}{P^{0}}. \end{array}$$

Then, the total changes of productive energy-related carbon emission can be expressed as

(7) ΔC=CT−C0=ΔCf+ΔCm+ΔCt+ΔCs+ΔCg+ΔCl+ΔCr+ΔCh+ΔCp,

where *α* = (*C*_*ij*_^*T*^ − *C*_*ij*_^0^)/(ln⁡*C*_*ij*_^*T*^ − ln⁡*C*_*ij*_^0^) and Δ*C*_*f*_, Δ*C*_*m*_, Δ*C*_*d*_, Δ*C*_*s*_, Δ*C*_*g*_, −Δ*C*_*l*_, Δ*C*_*r*_, Δ*C*_*h*_,  and Δ*C*_*p*_ are the contribution values to total carbon emission by the changes of carbon emission coefficient, energy mix, energy intensity, industrial structure, land economic output, land urbanization, urban area of per capita, population urbanization, and population size, respectively. *l* is the reciprocal of land urbanization rate, so the contribution values caused by changes of land urbanization are −Δ*C*_*l*_ after LMDI decomposition.

Carbon emission coefficient of different kinds of energy is generally treated as constant in the actual application. Therefore, in the decomposition model, Δ*C*_*f*_ = 0. The formula (7) can be simplified as

(8) ΔC=CT−C0=ΔCm+ΔCt+ΔCs+ΔCg+ΔCl+ΔCr+ΔCh+ΔCp.

#### 2.2.3. Decoupling Elasticity Decomposition Quantitative Model of Energy-Related Carbon Emission

Combining formula (8) with formula (3), the decoupling elasticity decomposition quantitative model of energy-related carbon emission is established as follows:

(9) Dt=ΔC/CΔGDP/GDP=ΔCC×GDPΔGDP=ΔC×GDPC×ΔGDP=(ΔCm+ΔCd+ΔCs+ΔCg+ΔCl+ΔCr+ΔCh+ΔCp)×GDPC×ΔGDP=ΔCm/CΔGDP/GDP+ΔCd/CΔGDP/GDP+ΔCs/CΔGDP/GDP+ΔCg/CΔGDP/GDP+ΔCl/CΔGDP/GDP+ΔCr/CΔGDP/GDP+ΔCh/CΔGDP/GDP+ΔCp/CΔGDP/GDP=Dm+Dd+Ds+Dg+Dl+Dr+Dh+Dp,

where *D*_*t*_ is the decoupling elasticity value of total energy-related carbon emission and economic growth and *D*_*m*_, *D*_*d*_, *D*_*s*_, *D*_*g*_, −*D*_*l*_, *D*_*r*_, *D*_*h*_, and *D*_*p*_ are the decoupling elasticity values of energy mix, energy intensity, industrial structure, land economic output, land urbanization, urban area of per capita, population urbanization, and population size, respectively.

### 2.3. Data Sources and Processing

The energy data used in this paper are quoted from* Energy Balance Sheet of Guangdong Province* in the* China Energy Statistical Yearbook* (1996–2012). Other data come from the* Statistical Yearbook of Guangdong Province* (1996–2012) and* Statistical Yearbook of china* (1996–2012) of the corresponding year. To eliminate the effect of price changes, we converted the GDP at current price to the GDP at constant price in the year 2000 by using indices of GDP (IGDP, preceding year = 100). The year 1995 is set as baseline year in LMDI method.

## 3. Results and Discussion

### 3.1. Analysis on Total Energy-Related Carbon Emission

**Figure 2:**
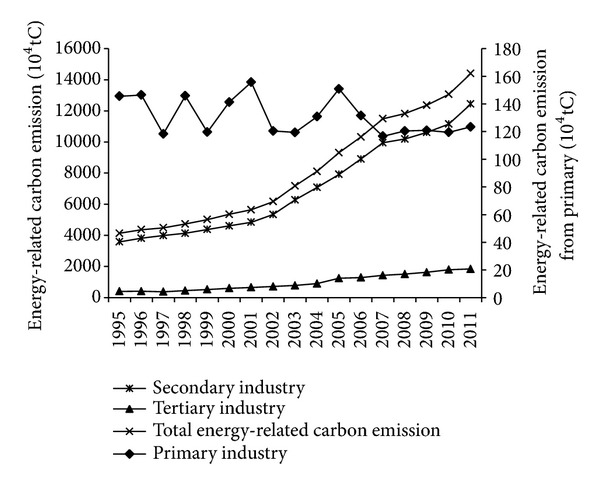
Change trends of energy-related carbon emission from 1995 to 2011.

The estimated results (Figure 2) show that the total energy-related carbon emissions in Guangdong province increased from 4129 × 10^4^ tC (tC, ton of Carbon) in 1995 to 14396 × 10^4^ tC in 2011, increased by 10267 × 10^4^ tC and the average annual growth rate is 8.12%. Among the three strata of industry, energy-related carbon emissions from the primary industry show decreasing trend, which fluctuate in a narrow range, decreasing from 146 × 10^4^ tC in 1995 to 123 × 10^4^ tC in 2011, and the average annual decline rate is 1.03%. The energy-related carbon emissions from the secondary industry and the tertiary industry both show increase trends, increasing from 3580 × 10^4^ tC and 403 × 10^4^ tC in 1995 to 12435 × 10^4^ tC and 1838 × 10^4^ tC in 2011, respectively, and the average annual growth rates are 8.09% and 9.94%, respectively. It is obvious that the secondary industry is the largest source of carbon emission, which accounts for more than 85% of the total energy-related carbon emission. The tertiary industry is the second largest source, which accounts for about 10% of the total energy-related carbon emission. The primary industry accounts for a small proportion and shows decline trend year by year.

### 3.2. Analysis on Decoupling Relationship between Energy-Related Carbon Emission and Economic Growth

Results of decoupling elasticity values and decoupling states of various decomposition factors can be seen in Table 3 and Figure 5.

**Figure 3:**
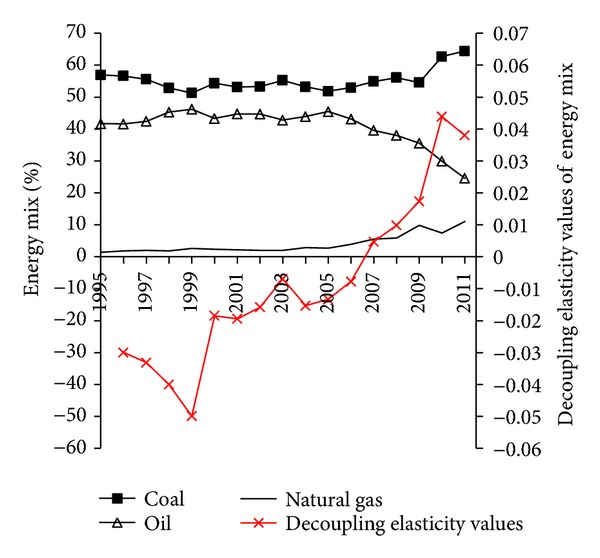
Energy mix and decoupling elasticity values of energy mix from 1995 to 2011.

The decoupling elasticity values of energy mix were negative in 1996–2006 and turned to positive in 2007–2011 (Figure 3), and the decoupling states turned into weak decoupling from strong decoupling. Mainly because the energy mix in Guangdong got some improvement during 1996–2006, the proportion of coal shows decline trend. The proportion of coal rose significantly since 2006, although the proportion of oil consumption in this period declined while the proportion of natural gas rose and the structure of high-carbon energy consumption with coal and oil as primary in Guangdong province has not improved greatly, especial since 2010 (Figure 3). This indicates that adjustment of energy mix in Guangdong is good for decoupling of energy-related carbon emission from economic growth in 1996–2006 but not beneficial to decoupling of energy-related carbon emission from economic growth in 2007–2011.

**Figure 4:**
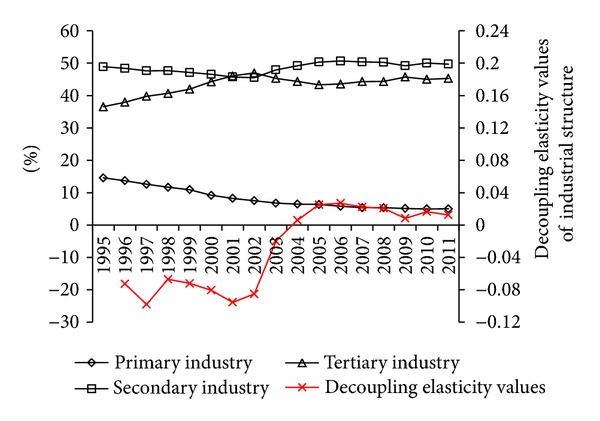
Industrial structure and decoupling elasticity values of industrial structure from 1995 to 2011.

The decoupling elasticity values of industrial structure turned into positive from negative in 2004, and the decoupling states turned into weak from strong. Mainly because the proportion of secondary industry increased while the proportion of tertiary industry reduced since 2002 (Figure 4). This indicates that the adjustment of industrial structure in Guangdong has not effectively reduced the carbon emission but promoted the carbon emission since 2004. It is good for decoupling of energy-related carbon emission from economic growth in 1996–2003 but not beneficial to decoupling of energy-related carbon emission from economic growth in 2004–2011.

As a kind of representative form of economic development level, the land economic output is closely related to the economic growth, so it is always in the expansive coupling state. Its decoupling elasticity values increase from 1.03 in 1996 to 1.17 in 2011 and land economic output is the first important inhibited factor for the decoupling of energy-related carbon emission from economic growth.

Energy intensity is the comprehensive index of technical progress for a country or region. During the research, the energy intensity in Guangdong reduces year by year, decreasing from 1.15 (tsce) per 10,000 yuans (RMB) in 1995 to 0.72 (tsce) per 10,000 yuans in 2011, which indicates that technical level in Guangdong has greatly improved. Decoupling elasticity values of energy intensity are always negative, which indicates that the energy intensity is the main motivator to realize the decoupling of energy-related carbon emission from economic growth.

Population size is always in the weak decoupling, but the changes of decoupling elasticity value for population size are smaller, indicating the relatively smaller influence of the population size on the decoupling relationship between energy-related carbon emission and economic growth in Guangdong.

**Figure 5:**
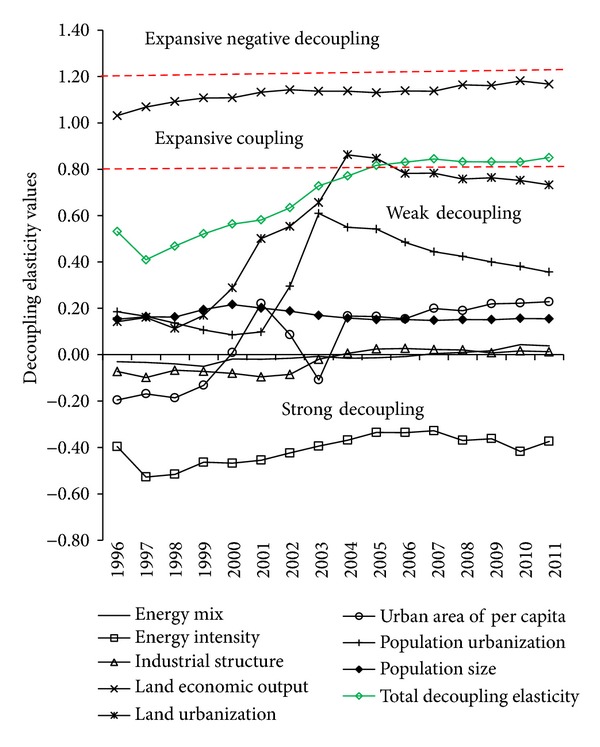
Change trends of each of decoupling elasticity values between energy-related carbon emission and economic growth from 1996 to 2011.

During the research, the decoupling elasticity values of land urbanization and population urbanization both show “inverted-N” trend (Figure 5), indicating that the decoupling states of both urbanization indexes are unstable and easily affected by external factors. But the turning point years of two “inverted-N” are different. The ascent stage of land urbanization is in 1998–2004 while population urbanization is in 2000–2003. In addition, the decoupling elasticity values of land urbanization higher than population urbanization, and the population urbanization always shows weak decoupling state in the whole research time, while the land urbanization shows weak decoupling state in the whole research time except in 2004-2005 when it shows expanded coupling state, which indicated that the land urbanization has more inhibiting effect than the population urbanization on the decoupling of energy-related carbon emission from economic growth during the research period.

The urban area of per capita is the result of combined action of land urbanization and population urbanization. Urban area of per capita increased year by year and indicates that development speed of the two urbanizations is uncoordinated. Decoupling elasticity values of the urban area of per capita were negative in 1996–1999 and turned to positive in 2000–2011 (except in 2003, may be influenced by SARS), and the decoupling states turned into weak decoupling from strong decoupling, which indicates that the increase of urban area of per capita is not beneficial to decoupling of energy-related carbon emission from economic growth.

The total decoupling elasticity values between energy-related carbon emission and economic growth in Guangdong province totally show increasing trend from 1996 to 2011, increased from 0.53 to 0.85, and the decupling state turned into expansive coupling from weak decoupling in 2005. Combining the above analysis on change trends of decoupling elasticity values and decoupling states of each factor, the total decoupling relationship between energy-related carbon emission and economic growth can be analyzed from the following two stages (see Figure 5).

*Stage I (1996–2004): Weak Decoupling Stage*. The energy-related carbon emission and economic growth show low carbon economy feature of “weak decoupling.” The decoupling elasticity value increases year by year (except the year of 1997), increased from 0.53 in 1996 (0.40 in 1997) to 0.77 in 2004. The decoupling elasticity value declined markedly in 1997, which was mainly attributed to the influence of Asia-Pacific financial crisis on economy and energy-related carbon emission since 1997. The financial crisis directly leads to the slow increase of economy in Guangdong, and the increasing speed of energy-related carbon emission is also affected in a certain degree, resulting in temporary decoupling intensive process between energy-related carbon emission and economic growth.

**Figure 6:**
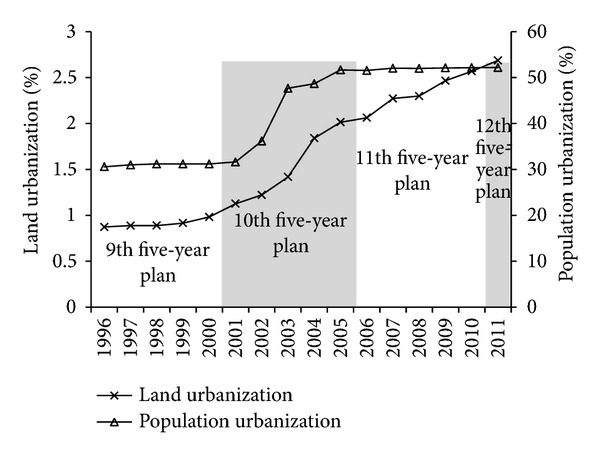
Change trends of urbanization in Guangdong from 1995 to 2011.

After 1998, with recovering economy and speeding up industrialization process, the economy and energy-related carbon emission in Guangdong both show rapidly increasing trend, the decoupling relations between them get weaker and weaker, and the features of the low carbon economy are not obvious increasingly. This situation is mainly affected by land urbanization. In the later period of the 9th five-year plan (1998–2000), land urbanization extended ahead of the population urbanization (Figure 6, land urbanization increased by 0.1% and population urbanization almost negative growth) and the decoupling elasticity values of land urbanization increased from 0.11 to 0.29. Land urbanization and population urbanization both speed up during the 10th five-year plan (2001–2005) (see Figure 6), which are related to the real estate investment, relative low price of housing, and more flexible land policies. Decoupling elasticity values of land urbanization and population urbanization increase from 0.50 and 0.10 in 2001 to 0.86 and 0.55 in 2004, respectively, which indicate that acceleration of both land urbanization and population urbanization in this period are not beneficial to the decoupling of energy-related carbon emission from economic growth. Although the changes of energy intensity resulted in the strong decoupling relationship between energy-related carbon emission and economic growth, the strong decoupling relationship is weakening (Figure 5) and could not offset the adverse effects of land economic output and urbanization. In addition to these, land economic output is the first important inhibited factor for the decoupling of energy-related carbon emission from economic growth during the research, but it changed a little, so it does not have much impact on the change of the total decoupling elasticity values. From what has been discussed above, land economic output and land urbanization are the main inhibited factors to decoupling of energy-related carbon emission from economy growth, but land urbanization plays a bigger role in change of the total decoupling elasticity values. Energy intensity is the main drive factor during 1996–2004.

*Stage II (2005–2011): Expansive Coupling Stage*. The decoupling relationship between energy-related carbon emission and economic growth turnd into expansive coupling from weak decoupling in stage I. But total decoupling elasticity values of energy-related carbon emission and economic growth do not rise continuously as in stage I, fluctuating in the range of 0.82–0.84 instead. That is, the energy-related carbon emission and economic growth in Guangdong do not realize “redecoupling” effectively and this is mainly attributed to the following two aspects.

One is the pressure of energy conservation and emission reduction. Our nation started to deploy the energy conservation and emission reduction in 2005. Guangdong positively responds to the call and formulates the target to drop the energy consumption per unit GDP by 16% during the 11th five-year plan compared to that in 2005. The work on energy conservation and emission reduction has achieved good results by eliminating lagging productive capacity and shutting down part of high energy consumption factories such as small thermal power and cement plant. Our government promised to reduce CO_2_ emission per unit GDP by 40%–45% in 2020 compared to that in 2005 in Copenhagen Climate Change Conference in 2009. All of these limit the rapid increase in carbon emission of Guangdong, combined with the rise of the technical level of Guangdong, and strong decoupling state of energy intensity is stronger (Figure 5), and all of above limit the redecoupling of energy-related carbon emissions from economic growth.

The other one is the influence of two urbanizations. Decoupling elasticity values of land urbanization and population urbanization both show decline trend since 2005 (Figure 5), mainly because the growth speed of land urbanization during the 11th five-year plan is slower compared with that during the 10th five-year plan, but still faster than population urbanization. Growth speed of population urbanization is nearly zero during the 11th five-year plan (Figure 6). Both of land urbanization and population urbanization play decisive roles in preventing continuously growth of total decoupling elasticity values of energy-related carbon emission and economic growth in stage II. We can see that slowing down the speed of both urbanizations is good for decoupling of energy-related carbon emission from economic growth. But development of urbanization is imperative in our country, and high quality urbanization should be population gathering and land saving and intensive use. Speed of land urbanization and population urbanization is obviously uncoordinated at this stage. The important reason is that with overdependence on land and real estate by economic development and local finance, the towns expand fast in space, and the farmlands are encroached by urbanization, but due to the restriction of household registration policy in Guangdong, many farmers cannot become real urban residents of modern city though their land are requisitioned for urbanization. This reflects that what the urbanization of Guangdong pursuit is still rapid expansion of space and spread development in this stage. So coordinating the development speed of land urbanization and population urbanization will have great significance to the construction of new urbanization in our country.

Under the combined action of the above two aspects, the energy-related carbon emission and economic growth maintain relative stable expansion coupling state from 2005 to 2011.

## 4. Conclusions and Policy Implication

### 4.1. Conclusions

Based on the extended Kaya identity and Tapio decoupling model, the decoupling elasticity decomposition quantitative model of energy-related carbon emission in Guangdong is established with the Logarithmic Mean Divisia Index (LMDI) method and influence factors of decoupling between carbon emissions and economic growth energy are decomposed into eight factors and urbanization factors are included into the decoupling model for the first time. Main results show that total production energy-related carbon emission in Guangdong shows increasing trend from 1995 to 2011, increase from 4128 × 10^4^ tC in 1995 to 14396 × 10^4^ tC in 2011. Decoupling elasticity values of energy-related carbon emission and economic growth show increasing trend from 1996 to 2011, and its decoupling state turns to expansive coupling in 2005–2011 from the weak decoupling in 1996–2004. Land economic output and energy intensity are the first inhibiting factor and first promoting factor to energy-related carbon emission decoupling from economic growth, respectively. The development speeds of land urbanization and population urbanization, especially land urbanization, play decisive roles in the change of decoupling elasticity values. Guangdong cannot realize decoupling of energy-related carbon emission from economic growth in a short time and there is a long way to go to implement low carbon province construction in Guangdong.

### 4.2. Policy Implication and Suggestions

There is a long way to go for Guangdong to realize decoupling of energy-related carbon emission from economic growth from the above analysis. Although Guangdong took many measures to carbon emissions reduction, for example, eliminating lagging productive capacity and shutting down part of high energy consumption factories such as small thermal power and cement plant in the 11th five-year plan period, these measures are not sustainable. The authors hold that there are four most effective measures to realize decoupling of energy-related carbon emission from economy growth for Guangdong according to analysis results and discussion in Section 3 and the current situation that Guangdong is facing.

(1)* Adjusting Energy Mix to Accelerate the Development of Low Carbon New Energy*. Its subtropical maritime climate characteristics make Guangdong rich in solar energy, wind energy, biomass energy, oceanic energy, and other new energy resources, and it is endowed with broad space and potential to develop and utilize low carbon energy sources. The biomass energy is an important renewable energy source, which has become the world's fourth-largest energy. The offshore area with eutrophication is an important advantage to develop the biomass energy for Guangdong. This can govern the red tide and acquire the energy and generate carbon sink. Therefore, Guangdong should pay more attention to the research on marine biomass energy and accelerate the progress of its development and utilization.

(2)* Adjusting the Industrial Structure to Accelerate Development of the Tertiary Industry*. The industry has been turn into heavy chemical industry since 2004, which has greatly promoted the development of economic in Guangdong. However, the industrial structure is not beneficial to reduce the carbon emission increasingly, and the current industrial structure level is not good for decoupling of carbon emission from economic growth. The development space of the tertiary industry is huge, so Guangdong can accelerate its development by developing the modern service industry to provide strong driving force for optimization and upgrading of industrial structure, taking education and tourism as a new economic growth point, providing guarantee for the development of the third industry from institutional, environmental, and the law. And finally improve the industrial structure level and realize the carbon emission reduction.

(3)* Coordinating the Development Speed of Land Urbanization and Population Urbanization*. The urbanization is imperative in our country, but high quality urbanization should be population gathering and land saving and intensive use. At present, there exist uncoordinated problems between development speed of the two urbanizations, land urbanization faster than population urbanization. The development speeds of land urbanization and population urbanization, especially land urbanization, play decisive roles in the change of decoupling elasticity values, so the speeds of the two urbanizations should be optimized properly by the following two measures. On the one hand, improve the utilization efficiency of land, and adopt tougher land red line and ecological line, makes the land use cannot be changed easily. On the other hand, formulate reasonable settling condition and regulate house price, makes it be conducive to the realization of high-quality urbanization and to the decoupling of carbon emission from economic growth.

(4)* Exploring Potential of Carbon Sink and Intensifying Carbon Sink Construction.* The total carbon emissions are still increasing due to the promotion of economic development and urbanization, and the emission reduction role of adjustment of energy mix and industrial structure cannot be played in the short time. Thus, exploring the carbon sink potentials is very important. It is an effective measure to plant the green manures in winter to absorb CO_2_ and reduce chemical fertilizer and improve soil and enhance land capacity. The carbon sink function of shrub land is stronger and it can increase the areas of shrub land through closing hill sides to facilitate afforestation [38].

## Figures and Tables

**Table 1:** Carbon emission coefficients of different kinds of energy.

Energy type	Net calorific value (TJ/10^3^t)	Carbon content (t/TJ)	Carbon emission coefficients (tC/t)
Raw coal	20.7	26.6	0.55
Washed clean coal	28.2	25.8	0.73
Other types of washed coal	28.2	25.8	0.73
Briquettes	20.7	26.6	0.55
Coke	28.2	29.2	0.82
Coke-oven gas			0.20
Other gases			0.20
Other coking products	28.2		0.82
Natural gas			0.44
Crude oil	42.3	20.0	0.85
Gasoline	44.3	18.9	0.84
Kerosene	43.8	19.6	0.86
Diesel oil	43.0	20.2	0.87
Fuel oil	40.4	21.1	0.85
Liquefied petroleum gas	47.3	17.2	0.81
Refinery gas	49.5	15.7	0.78
Other petroleum products	40.2	20.0	0.80

Notes: (1) The unit of carbon emission coefficients of “Coke-oven gas,” “other gases,” and “natural gas” is “ton carbon/ton standard coal equivalent” or “tC/tsce.” Carbon emission coefficient of natural gas comes from reference [35] and carbon emission coefficients of “coke-oven gas” and “other gases” are calculated according to the relationship between their calorific value and natural gas. (2) The unit of other energy's carbon emission coefficient is “ton C/ton” or “tC/t.” It represents carbon emission from one tone physical quantity energy. Carbon emission coefficient = net calorific value × carbon content, net calorific value, and carbon content come from *2006  IPCC  Guidelines  for  National  Greenhouse  Gas  Inventories* [36]. Carbon content per unit coal is higher than oil, but its net calorific value is lower than that of oil, resulting in the carbon emission coefficient of coal being lower than for oil. We reference here the paper [37].

**Table 2:** Eight decoupling states divided by Tapio (2005) [15].

Decoupling elasticity values (*D*_*t*_)	Δ*C*/*C*	ΔGDP/GDP	Decoupling states
*D*_*t*_ < 0	<0	>0	Strong decoupling
0 ≤ *D*_*t*_ < 0.8	>0	>0	Weak decoupling
0.8 ≤ *D*_*t*_ ≤ 1.2	>0	>0	Expansive coupling
*D*_*t*_ > 1.2	>0	>0	Expansive negative decoupling
*D*_*t*_ < 0	>0	<0	Strong negative decoupling
0 ≤ *D*_*t*_ < 0.8	<0	<0	Weak negative decoupling
0.8 ≤ *D*_*t*_ ≤ 1.2	<0	<0	Recessive coupling
*D*_*t*_ > 1.2	<0	<0	Recessive decoupling

**Table:** (a)

	Energy mix		Energy intensity		Industrial structure		Land economic output	
	Value *D*_*m*_	State of decoupling	Value *D*_*d*_	State of decoupling	Value *D*_*s*_	State of decoupling	Value *D*_*g*_	State of decoupling
1996	−0.03	SD	−0.40	SD	−0.07	SD	1.03	EC
1997	−0.03	SD	−0.53	SD	−0.10	SD	1.07	EC
1998	−0.04	SD	−0.52	SD	−0.07	SD	1.09	EC
1999	−0.05	SD	−0.46	SD	−0.07	SD	1.11	EC
2000	−0.02	SD	−0.47	SD	−0.08	SD	1.11	EC
2001	−0.02	SD	−0.46	SD	−0.10	SD	1.13	EC
2002	−0.02	SD	−0.42	SD	−0.09	SD	1.14	EC
2003	−0.01	SD	−0.39	SD	−0.02	SD	1.14	EC
2004	−0.02	SD	−0.37	SD	0.01	WD	1.14	EC
2005	−0.01	SD	−0.34	SD	0.03	WD	1.13	EC
2006	−0.01	SD	−0.34	SD	0.03	WD	1.14	EC
2007	0.00	WD	−0.33	SD	0.02	WD	1.14	EC
2008	0.01	WD	−0.37	SD	0.02	WD	1.16	EC
2009	0.02	WD	−0.36	SD	0.01	WD	1.16	EC
2010	0.04	WD	−0.42	SD	0.02	WD	1.18	EC
2011	0.04	WD	−0.37	SD	0.01	WD	1.17	EC

**Table:** (b)

	Land urbanization		Urban area of per capita		Population urbanization		Population size		Total decoupling elasticity	
	Value −*D*_*l*_	State of decoupling	Value *D*_*r*_	State of decoupling	Value *D*_*h*_	State of decoupling	Value *D*_*p*_	State of decoupling	Value *D*_*t*_	State of decoupling
1996	0.14	WD	−0.20	SD	0.19	WD	0.15	WD	0.53	WD
1997	0.16	WD	−0.17	SD	0.17	WD	0.16	WD	0.41	WD
1998	0.11	WD	−0.19	SD	0.14	WD	0.16	WD	0.47	WD
1999	0.17	WD	−0.13	SD	0.11	WD	0.19	WD	0.52	WD
2000	0.29	WD	0.01	WD	0.09	WD	0.22	WD	0.56	WD
2001	0.50	WD	0.22	WD	0.10	WD	0.20	WD	0.58	WD
2002	0.55	WD	0.09	WD	0.30	WD	0.19	WD	0.63	WD
2003	0.66	WD	−0.11	SD	0.61	WD	0.17	WD	0.73	WD
2004	0.86	EC	0.17	WD	0.55	WD	0.16	WD	0.77	WD
2005	0.85	EC	0.16	WD	0.54	WD	0.15	WD	0.82	EC
2006	0.78	WD	0.15	WD	0.48	WD	0.15	WD	0.83	EC
2007	0.78	WD	0.20	WD	0.44	WD	0.15	WD	0.84	EC
2008	0.76	WD	0.19	WD	0.42	WD	0.15	WD	0.83	EC
2009	0.76	WD	0.22	WD	0.40	WD	0.15	WD	0.83	EC
2010	0.75	WD	0.22	WD	0.38	WD	0.16	WD	0.83	EC
2011	0.73	WD	0.23	WD	0.36	WD	0.15	WD	0.85	EC

Notes: SD represents strong decoupling; WD represents weak decoupling; EC represents expansive coupling.
